# Metaproteomics reveals potential mechanisms by which dietary resistant starch supplementation attenuates chronic kidney disease progression in rats

**DOI:** 10.1371/journal.pone.0199274

**Published:** 2019-01-30

**Authors:** Boris L. Zybailov, Galina V. Glazko, Yasir Rahmatallah, Dmitri S. Andreyev, Taylor McElroy, Oleg Karaduta, Stephanie D. Byrum, Lisa Orr, Alan J. Tackett, Samuel G. Mackintosh, Ricky D. Edmondson, Dorothy A. Kieffer, R. J. Martin, Sean H. Adams, Nosratola D. Vaziri, John M. Arthur

**Affiliations:** 1 Department of Biochemistry and Molecular Biology, University of Arkansas for Medical Sciences, Little Rock, AR, United States of America; 2 Department of Biomedical Informatics, University of Arkansas for Medical Sciences, Little Rock, AR, United States of America; 3 Proteomics Core Facility, University of Arkansas for Medical Sciences, Little Rock, AR, United States of America; 4 Department of Nutrition, University of California, Davis, CA, United States of America; 5 Arkansas Children's Nutrition Center and Department of Pediatrics, University of Arkansas for Medical Sciences, Little Rock, AR, United States of America; 6 Division of Nephrology, University of California, Irvine, CA, United States of America; 7 Division of Nephrology, University of Arkansas for Medical Sciences, Little Rock, AR, United States of America; Southern Illinois University School of Medicine, UNITED STATES

## Abstract

**Background:**

Resistant starch is a prebiotic metabolized by the gut bacteria. It has been shown to attenuate chronic kidney disease (CKD) progression in rats. Previous studies employed taxonomic analysis using 16S rRNA sequencing and untargeted metabolomics profiling. Here we expand these studies by metaproteomics, gaining new insight into the host-microbiome interaction.

**Methods:**

Differences between cecum contents in CKD rats fed a diet containing resistant starch with those fed a diet containing digestible starch were examined by comparative metaproteomics analysis. Taxonomic information was obtained using unique protein sequences. Our methodology results in quantitative data covering both host and bacterial proteins.

**Results:**

5,834 proteins were quantified, with 947 proteins originating from the host organism. Taxonomic information derived from metaproteomics data surpassed previous 16S RNA analysis, and reached species resolutions for moderately abundant taxonomic groups. In particular, the *Ruminococcaceae* family becomes well resolved–with butyrate producers and amylolytic species such *as R*. *bromii* clearly visible and significantly higher while fibrolytic species such as *R*. *flavefaciens* are significantly lower with resistant starch feeding. The observed changes in protein patterns are consistent with fiber-associated improvement in CKD phenotype. Several known host CKD-associated proteins and biomarkers of impaired kidney function were significantly reduced with resistant starch supplementation. Data are available via ProteomeXchange with identifier PXD008845.

**Conclusions:**

Metaproteomics analysis of cecum contents of CKD rats with and without resistant starch supplementation reveals changes within gut microbiota at unprecedented resolution, providing both functional and taxonomic information. Proteins and organisms differentially abundant with RS supplementation point toward a shift from mucin degraders to butyrate producers.

## Introduction

Recent studies point to gut microbiome dysbiosis as one of the key contributors to the progression of chronic kidney disease (CKD) and its complications [1–3]. During the course of CKD, gut dysbiosis increases and compromises the intestinal epithelial barrier, leading to leakage of microbial-derived toxins into the bloodstream and resulting in increased inflammation that may further exacerbate CKD [2]. One suggested contributor to the dysbiosis is increased urea in intestinal fluids. Consequently, the urease-containing species proliferate in the gut, leading to damage of the epithelial barrier. Indeed, the CKD-associated microbiota have been characterized by an increase in bacterial species encoding for urease and uricase, and indole- and p-cresol producing enzymes, and depletion of microbes expressing short-chain fatty acid-forming enzymes [4].

Currently, CKD patients are often prescribed a diet that contains low quantities of fiber in order to limit the intake of potassium and avoid cardiac arrhythmias. However, in various models it has been shown that certain fibers can promote gut health and function, by increasing a gut microbiota population that dampens gut permeability and limits damage to the mucus layer caused by utilization of host glycans. Since CKD is a pro-inflammatory condition, and kidney damage may be exacerbated under conditions of gut microbiota dysbiosis, it is worth considering if increasing dietary fiber could help limit CKD complications and improve kidney function. One potential candidate to supplement a CKD diet is high-amylose maize-resistant starch type 2 (HAMRS2), a prebiotic which is metabolized by the gut microbes and has been shown to improve outcomes in a rat model of CKD [5, 6]. A previous study using taxonomic analysis characterized microbiome-related changes caused by resistant starch (RS) supplementation in CKD rats [5]. Microbiome and metabolomics data were correlated to identify potential metabolic pathways impacted by gut bacteria and linked to improvements in kidney function. In summary the previous study [5] provided strong evidence that resistant starch-induced microbiome shifts results in reduced inflammation and protection of the gut epithelial barrier.

Earlier studies in healthy humans, animals, and *in vitro* models showed increased levels of *Bifidobacterium*, *Ruminococcus*, *Lactobacillus*, *Bacteroides*, *Eubacterium*, *Allobaculum*, and *Prevotella* upon dietary supplementation of RS [7–11]. Some of these organisms, e.g. *Ruminococcus bromii*, have been shown to contain genes for starch utilization and are proven direct degraders of RS [12–14]. The organisms that increase in abundance upon RS supplementation are feeding on mono- and oligo- saccharides derived from RS-degradation by the direct degraders [12]. *In vitro* stable-isotope probing followed by 16S rRNA sequencing of metabolically labeled RNA further validated that many of these bacteria utilize RS [15]. Similar trends–showing increases in RS degraders and utilizers upon supplementation with RS in the rat model of CKD were observed by Kieffer et al. [5].

To gain further insight into the mechanism of RS action, we employed a metaproteomics analyses of the samples derived from the CKD rat model described previously [5, 16]. These analyses are capable of characterizing a complex protein mixture from an environmental sample.

## Methods

**Fig 1:**
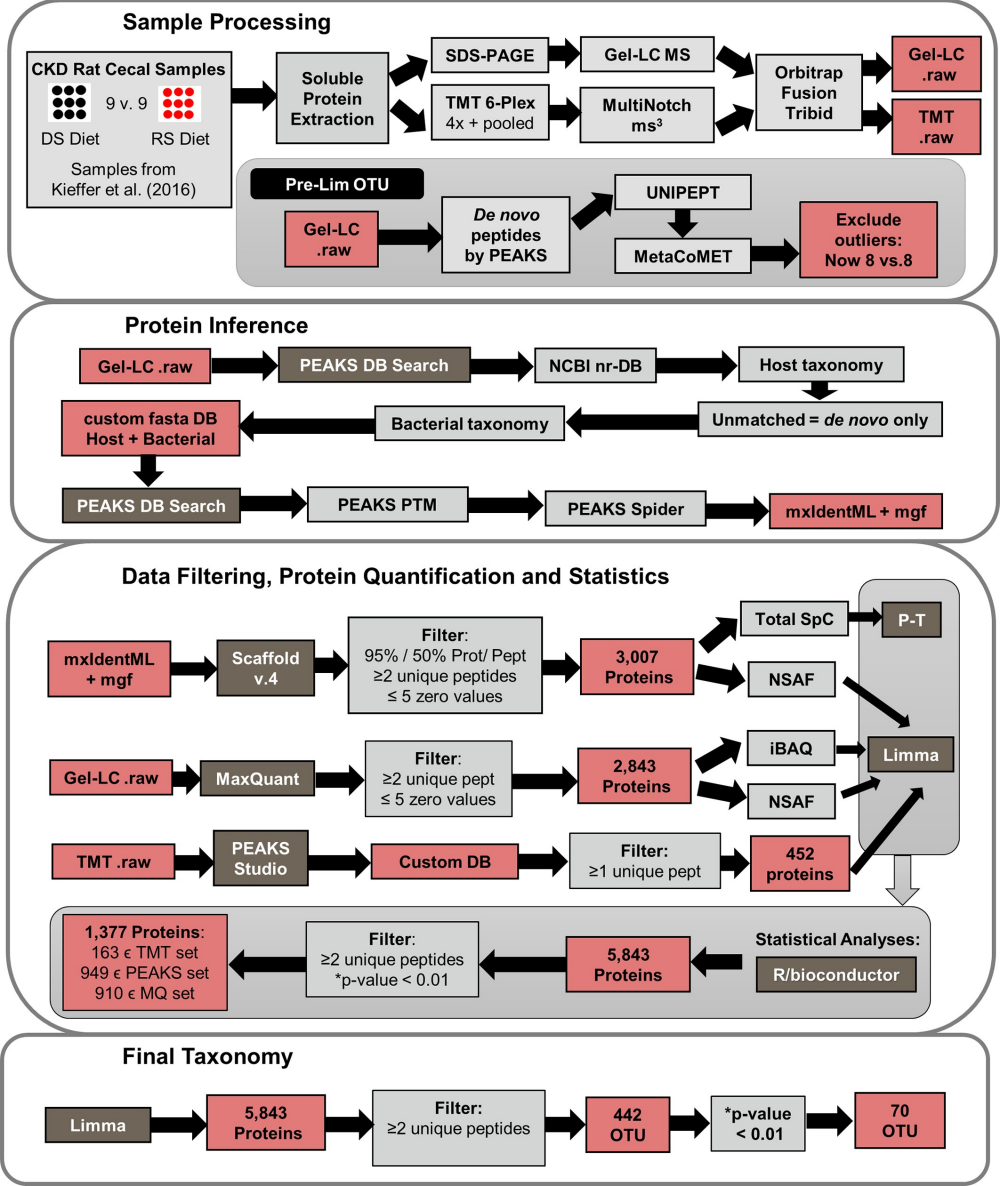
Experimental and analytical work flow. The metaproteomics data-analysis pipe-line consists of sample processing, mass-spectrometry data acquisition and preliminary analysis (*sample processing panel*). *Protein inference panel* shows the software tools used to arrive at the identified proteins list. *Data Filtering*, *Protein Quantification and Statistics panel* shows procedures and tools used to arrive at the final quantified proteins list. *Final Taxomy panel* shows derivation of final taxonomic groups (operational taxonomic units, OTUs). Individual software tools, analytical, and statistical methods used are shown in *grey-shaded* boxes. Intermediate files and resulting numbers of sequences are shown in *pink-shaded* boxes.

To characterize differences in metaproteome composition between RS-fed rats with CKD (CKD-RS), and the CKD rats fed with a host-digestible starch (CKD-DS) we employed a combination of different quantitative mass spectrometry-based proteomics techniques—absolute intensity-based quantification (iBAQ), spectral counting and tandem mass tag (TMT) labeling. **Fig 1** summarizes the overall experimental and analytical workflow and the methods used.

### Animals and diets

We used cecal content samples obtained at the time of sacrifice in the previous study by Vaziri, *et al*. Animals, housing conditions, and diets have been previously described [16]. Briefly, 10-wk-old male Sprague-Dawley rats were purchased from Harlan Laboratories (Indianapolis, IN) and fed powdered chow (no. 2020X, Harlan Laboratories) containing 0.7% adenine for 2 weeks to induce CKD. Rats were then randomized to receive semi-purified pelleted diets supplemented with 59% by weight with either the digestible starch, amylopectin (low fiber), or indigestible starch, HAMRS2 (Hi-Maize 260, Ingredion, Westchester, IL), for 3 weeks (n = 9 rats/group). Isocaloric diets were prepared by Harlan Laboratories containing 14.5% protein, 66.9% carbohydrate and 18.6% fat. All animals were provided free access to food and water. Rats were placed in metabolic cages for 24-h urine collection. They were then anesthetized [ketamine (50 mg/kg) plus xylazine (4 mg/kg ip)] and euthanized via cardiac exsanguination between 8:00–11:00 AM. Blood was collected and left at room temperature for 30 min to clot, serum was then collected, frozen on dry ice, and stored at -70°C until processed. Cecal contents were removed, frozen on dry ice, and stored at -70°C until processed. Hydration of cecal contents was determined by weighing a frozen aliquot and then oven drying overnight at 100°C; the sample was allowed to cool and weighed twice to ensure a constant weight was obtained. All experiments were approved by the Institutional Committee for the Use and Care of Experimental Animals of the University of California (Irvine, CA). These animals and samples of their cecal content were used in the previous publication by Kieffer et al. [3]. In the current paper we analyze these samples via novel metaproteomics method. We preserved the original enumeration of animals as in Kieffer et al. [3] with animals 7 to 15 being the DS-fed group and animals 16 to 24 being the RS-fed group.

### Soluble protein extraction

In a cold room (4°C), 500 μl of PBS with protease inhibitor cocktail (ThermoFisher, Waltham, MA USA) was added to the 100 mg of the frozen cecal content and vortexed vigorously for 5 min followed by sonication for 5 min using Bioruptor (Diagenode, Denville, NJ). Bioruptor settings: High, 30 second on/off cycles. The lysates were centrifuged at 20,000 x g for 30 min. Pellets were saved at -80°C for future studies of membrane proteins. The supernatant (soluble proteins) were collected and protein concentration was determined using BCA assay (ThermoFisher). Reproducibility of the extraction has been evaluated by total de novo peptides identified and matched using the online metaproteomics tool Unipept [17]. Bacterial-to-host proteins ratio has been used as an extraction/sample quality criteria.

20 μg of protein for each sample was resolved on a 4–20% Tris-Gly gel. The gels lanes were cut in 24 pieces, in-gel digested and analyzed on the Thermo’s Orbitrap-Tribrid-Fusion instrument at the UAMS Proteomics Core. Spectral counting and tandem mass tags (TMTs) were used in parallel for protein quantification.

### TMT-labeling

The samples were labeled with TMT-126, 127, 128, 129, 130, and 131 tandem mass tags using the ThermoFisher TMT6plex kit. The samples were prepared in four independent TMT batches. In the first batch we mixed samples 7, 8, 16, 17, 18, and a pooled mixture of all 18 samples. The second batch contained samples 9, 10, 11, 19, 20, and the pooled sample. The third batch contained samples 12, 13, 21, 22, and the pool sample (skipped label 129). The fourth batch contained samples 14, 15, 23, 24, and the pool sample (skipped label 129). The isobaric tags have an identical chemical structure and same total mass but with labile parts under collision-activated dissociation (CID), known as the reporter ions [18]. The reporter ions are detected at distinct m/z. Therefore, by multiplexing the samples labeled with different TMT tags, we can determine both the identity and relative abundance of peptide pairs simultaneously [19]. Samples were prepared for TMT labeling by first diluting 100 μg of each protein lysate to 100μl in 50mM TEAB. The samples were reduced in 200 mM Tris [2-carboxyethyl] phosphine (Thermo) at 55°C for 1 hour. Next, the samples underwent alkylation in 50 mM iodoacetamide (Sigma-Aldrich) for 30 min. Proteins were precipitated by adding 100% (w/v) trichloroacetic acid (TCA) to a final concentration of 20% (v/v), incubating at -20°C for 1 hour, then centrifuging in a microfuge for 15 minutes at 4°C. The samples were washed in 20% TCA, 100% acetone (cold), and in 80% acetone (cold) in order to remove any primary amines. After washing, samples were resuspended in 100 ul of 50mM TEAB with 1 μg/ul of trypsin and incubated at 37°C overnight. After protein digestion, the samples were labeled following the TMT6plex labeling protocol. The samples were mixed at a 1:1:1:1:1:1 ratio. We first tested a 1 μg mixing of each sample by mass spectrometry and used median reporter ion intensity values to adjust mixing amounts.

### Basic HPLC and multinotch

The following two buffers were used for the basic HPLC: *Buffer A*, 0.1% formic acid, 0.5% acetonitrile; *Buffer B*, 0.1% formic acid, 99.9% acetonitrile. Both buffers adjusted to pH 10 with ammonium hydroxide for offline separation. Tryptic peptides were separated into 36 fractions on a 100 x 1.0 mm Acquity BEH C18 column (Waters) using an UltiMate 3000 UHPLC system (Thermo) with a 40 min gradient from 99:1 to 60:40 buffer A:B ratio under basic pH conditions, and then consolidated into 12 super-fractions. Each super-fraction was then further separated by reverse phase Jupiter Proteo resin (Phenomenex) on an in-line 200 x 0.075 mm column using a nanoAcquity UPLC system (Waters). Peptides were eluted using a 60 min gradient from 97:3 to 67:33 buffer A:B ratio. Eluted peptides were ionized by electrospray (2.15 kV) followed by mass spectrometric analysis on an Orbitrap Fusion Tribrid mass spectrometer (Thermo) using multi-notch MS^3^ parameters [20]. MS data were acquired using the FTMS analyzer in top-speed profile mode at a resolution of 240,000 over a range of 375 to 1500 m/z. Following CID activation with normalized collision energy of 35.0, MS/MS data were acquired using the ion trap analyzer in centroid mode over a range of 400–2000 m/z. Using synchronous precursor selection, up to 10 MS/MS precursors were selected for HCD activation with normalized collision energy of 65.0, followed by acquisition of MS^3^ reporter ion data using the FTMS analyzer in profile mode at a resolution of 60,000 over a range of 100–500 m/z.

### De novo peptide sequencing

Files that were acquired on the Orbitrap Fusion Tribrid mass spectrometer (in “.raw” format) were submitted to the *de novo* sequencing using PEAKS Studio v. 8 (Bioinformatics Solutions, Waterloo, ON, Canada). The following parameters were used for the data refinement: *Merge Scans*–left unchecked; *Correct Precursor–*mass only; *Filter Scans*–unchecked. The following parameters were used for the de novo sequencing: *Parent Mass Error Tolerance*– 5 ppm; *Fragment Mass Error Tolerance*– 0.5 Da; *Enzyme*–Trypsin; *Fixed Modifications–*Carbamidomethylation (C); *Variable Modifications*–Oxidation (M), Deamidation (NQ); Max Variable PTM Per Petpide– 3; *Report # Peptides*– 5.

### Preliminary taxonomy analysis and exclusion of outliers

**Fig 2:**
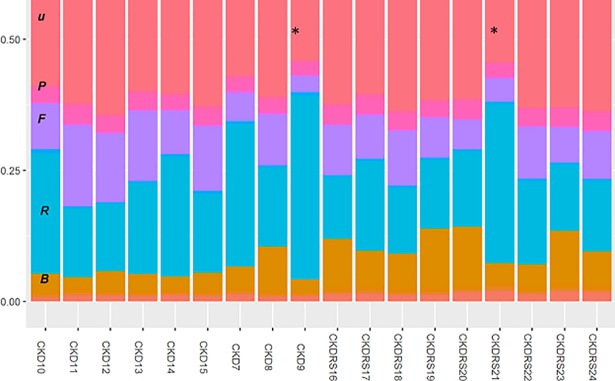
Taxonomy analysis at the *phylum level* using de novo peptides. De Novo peptides identified by PEAKS (ALC% score above 80) were exported into UniPept. UniPept output was converted to BIOME file format using in-house written R-script and further plotted using MetaCoMET online tool ([23], https://omictools.com/metagenomics-core-microbiome-exploration-tool-tool). * *Samples 9 and 21*: in the subsequent analysis these samples were proved to be outliers based on total host-to-bacterial protein ratios and excluded from quantification. Major groups are color-coded as followed (labels are shown for the CKD10 sample): ***u***–unassigned, hot-pink; ***P***–proteobacteria, pink; ***F–***fermicutes, purple; ***R***–chordata (rat), cyan; ***B***–bacteroidetes, light brown.

De novo peptides identified by PEAKS [21] were filtered using the average local confidence score (ALC%), and the peptides with ALC% above 80 were submitted to the online metaproteomics tool UniPept [17, 22]. In the Unipept/metaproteomics analysis tab, the following parameters were set: Equate I and L—checked, Filter duplicate peptides–unchecked, Advanced missed cleavage handling–checked. The taxonomy information was visualized using a tree diagram provided by UniPept (**S1 Fig**) and using MetaCoMET (Metagenomics Core Microbiome Exploration Tool [23], **Fig 2**). For the visualization in MetaCoMET, taxonomy tables were downloaded from UniPept and converted to BIOME files using in-house R-script (available upon request). Ratio between eukaryotic and bacterial proteins was calculated using numbers of spectral counts.

Samples derived from animals 9 and 21 had the most extreme host-to-bacterial proteins ratios (**Fig 2**, **S1 and S2 Figs**). In the subsequent hierarchical clustering analysis, these samples clustered together in a group separate from the two phenotypes. For these reasons samples 9 and 21 were excluded from the final dataset.

### Peptide identification and protein inference using database search

Multi-step database search strategy was employed in PEAKS Studio as follows: The default mode of FDR estimation in PEAKS using fused decoy strategy (which is compatible with the multi-step searches [24] was used. NCBI_nr protein database (downloaded on 08/29/2016, 74,510,638 total entries) was searched using *Rattus norvegicus* as a taxonomic filter, (80,472 non-redundant protein entries searched, Search 1). The results of the PEAKS DB Search 1 were filtered using 1% false discovery rate for peptide-to-spectrum matches (corresponding average -10lgP ~25 across samples) and minimum 1 unique peptide per protein. Unmatched *de novo* tags from the Search 1 were further searched against NCBI_nr protein database using Bacterial taxonomic filter (64,001,258 non-redundant protein entries searched, Search 2). Fasta protein sequences from the two searches, for all 18 rat cecum samples, unfiltered (combined peptide-to-spectrum FDR 5%, 0 unique peptides allowed), were combined into a custom fasta database for the final search (~75,000 protein sequences in the final database). Contribution from other taxonomies (e.g. *archaea*, *viruses*, *green plants and fungi–***S1 Fig***)* was deemed negligible, and was not used in the downstream analyses. The original lists of de novo peptides were re-searched using this custom fasta database using PEAKS DB, PEAKS PTM, and PEAKS Spider searches. The final SPIDER results were filtered using 1% false discovery rate for peptide-to-spectrum matches (corresponding average -10lgP ~20 across 18 samples) and minimum 2 unique peptides. The results were exported as mzIdentML files along with spectral data in mascot generic format for the downstream analyses in Scaffold v. 4.

Database search and Taxonomic analysis with MaxQuant and Andromeda and iBAQ quantification was performed as follows. The “raw” files obtained during the Gel-LC-MS/MS experiment were also searched by MaxQuant/Andromeda search engine, in addition to PEAKS, with subsequent quantification using NSAF and iBAQ (Absolute Label-Free Protein Quantification) methods. The following parameters were set for the MaxQuant search: Fixed modifications Carbamidomethyl (C); Decoy mode revert; Include contaminants True; Top MS/MS peaks per 100 Da. (FTMS) 12; MS/MS deisotoping (FTMS) True; Top MS/MS peaks per 100 Da. (ITMS) 8; PSM FDR 0.01; Protein FDR 0.01; Site FDR 0.01; Min. peptide Length 7; Min. score for modified peptides 40; Use only unmodified peptides and True Modifications included in protein quantification Oxidation (M);Acetyl (Protein N-term); Peptides used for protein quantification Razor. For the full set of MaxQuant parameters see supplemental methods. Spectral counts were normalized using NSAF method–weighing by protein length, normalizing to the total sum per sample, and taking log transform. The transformation and imputation of missing values was performed in Perseus statistical suite. Imputed values were drawn from a normal distribution, with a width of 30% of the valid data and shifted by 2 standard deviation from the mean value of the valid data. To define differentially abundant proteins, moderated T-test (limma Bioconductor package) was performed on the transformed and imputed values. iBAQ values were processed in a similar fashion: relative iBAQ values were calculated by divided by a sample sum followed by log2 transformation and imputation of the missing values in Perseus (same parameters for imputation as with the NSAF method). As with the NSAF method, differential abundance was established using moderated T-test (limma package) applied to the log-transformed and imputed dataset. As a result, 2,842 proteins were quantified. Taxonomic analysis was performed in exactly the same way as with PEAKS-derived proteins.

### Protein and taxonomic unit quantification using TMT labeling

PEAK Studio v. 8 with a quantitative module was used to analyze the TMT-labeled multiplexed experiments. Default parameters for the TMT MultiNotch MS3 analysis were selected and each of the four of the TMT-mixings was analyzed separately. Custom fasta database derived from the Gel-LC analysis was used. 1% peptide-to-spectrum FDR and 2 unique peptides per proteins were set as filters. Final data assembly to included all TMT-mixings, and ratios of normalized summed reporters intensities to pooled samples were recalculated in Microsoft Excel and exported into R for the downstream analysis. Custom fasta database derived from the Gel-LC analysis was used. 549 proteins were quantified using TMT method. *limma* Bioconductor package [25] was used as in the previous section to establish significance (p adjusted <0.05) for proteins and taxonomic units. 149 taxonomic units were identified in total using TMT method, with 45 established as significant.

### Data sharing

The mass spectrometry proteomics data have been deposited to the ProteomeXchange Consortium via the PRIDE [26] partner repository with the dataset identifier PXD008845 and 10.6019/PXD008845

### Statistical methods

For protein quantification, Scaffold v. 4 with quantitation module (Proteome Software) was used. Data and the custom fasta database were exported from PEAKS into Scaffold as mzIdentML and mascot generic format files. Scaffold-derived normalized spectrum abundance factors (NSAF [27]) values and Total Unique spectral counts were used for the statistical analysis in R/bioconductor [28]. Taxonomic information from the NCBI-nr database (downloaded Oct. 16, 2016) was used to derive taxonomic units. Sum of spectral counts matching a given taxonomic unit, weighted by a total spectral count per a given replicate, was used as a measure of abundance for that taxonomic unit. Moderated *t*-test (limma package, Bioconductor) was used to establish differential abundance of taxonomic units. Benjamini Hochberg correction for multiple testing was enforced both for protein and taxonomic unit quantification. Heatmaps and hierarchical clustering of spectral count and TMT datasets were performed using the Bioconductor package ComplexHeatmap [29]. PCA analysis was performed using base R.

## Results and discussion

We used cecal content samples from a previous study in which treatment with dietary resistant starch attenuated the progression of chronic kidney disease in a rat model of adeninine induced renal failure [16]. In the current paper we employed metaproteomics to assess the effect of resistant starch on gut content microbial and host proteins in a chronic kidney disease rat model. For the simultaneous analysis of host and microbial proteins we combined several distinct proteomics pipelines and experimental platforms. This strategy allowed us to overcome specific biases characteristics of different pipelines and to characterize host and microbial proteins to the fullest extent.

### Metaproteomics protein signature of cecal contents from rats fed resistant starch or digestible starch diets

**Fig 3:**
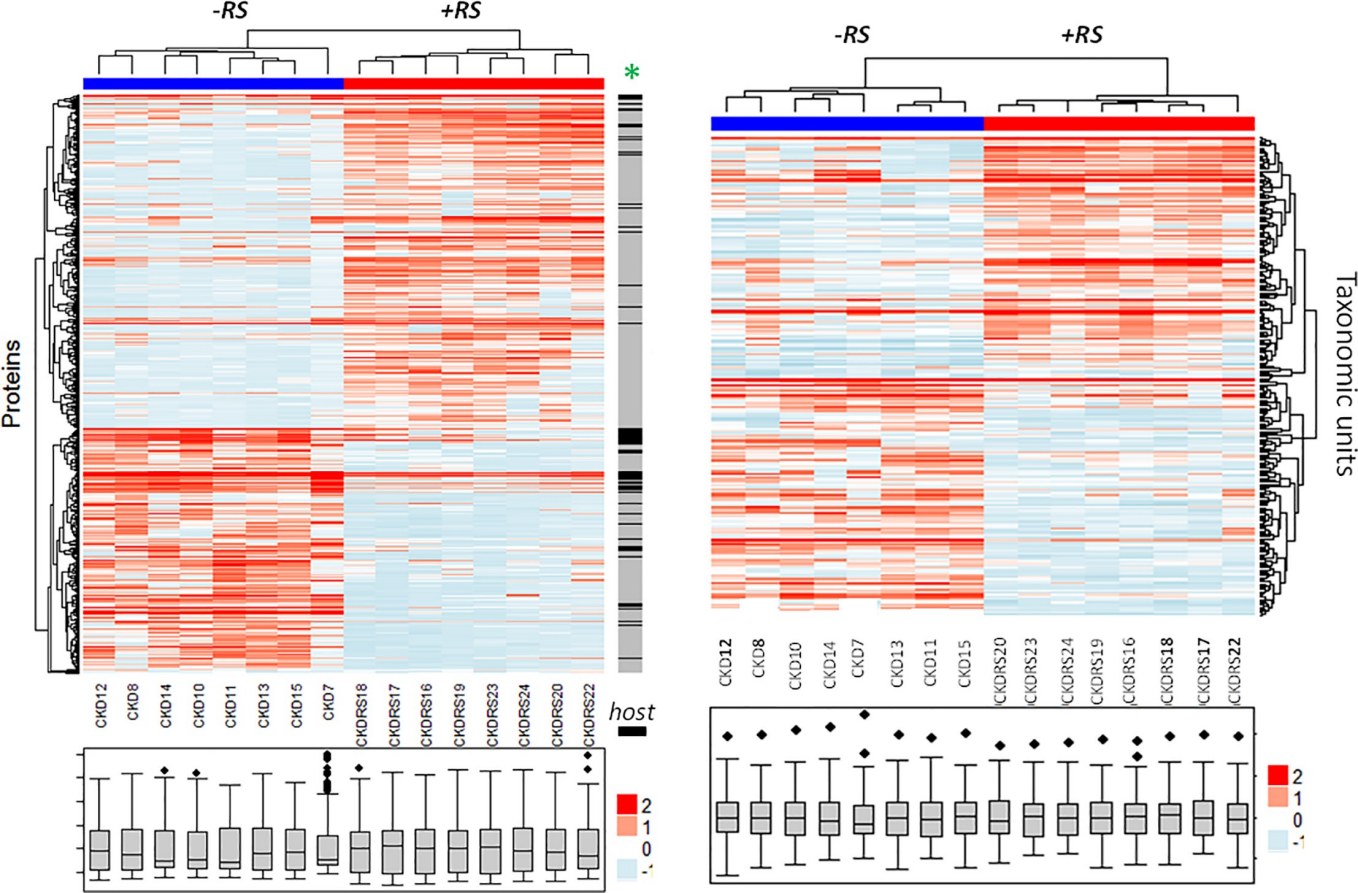
The phenotype induced by resistant starch in chronic kidney disease (CKD) rats is well-defined by metaproteomics data. *Left heatmap*, cecal content protein abundance values were averaged across three different labeling-free methods: PEAKS spectral counting, MaxQuant spectral counting and intensity based-quantification. The averaged values were log-transformed and normalized and plotted using ComplexHeatmaps R/Bioconductor package. 506 proteins, deemed significant by MaxQuant and by PEAKS are shown (p<0.01). CKD and CKDRS phenotypes are indicated by *blue* and *red* colors on the top heatmap annotation bar. Distribution properties are shown in BOX plots for each of the animals at the bottom, indicating similar distributions between different animals. The raw annotation bar is adjacent to the right edge of the heatmap–“*host”* indicates rat proteins by *black* color, and microbial proteins by *grey* color. *Right heatmap*, taxonomic units abundance values derived from the protein data were log-transformed and normalized and plotted using ComplexHeatmaps. Significant taxonomic groups (p <0.05) are shown.

Overall, our approach allowed us to identify 9,386 unique proteins sequences across all experimental samples and conditions (1% false discovery rate for peptide-to-spectrum matches and minimum 2 unique peptides, **S3 Fig**). Combining complementary quantification methods (TMT, PEAKS, and MaxQuant) and requiring that to be quantified, a protein has to have at least 3 non-zero abundance values in either ithe CKD-DS or CKD-RS group, out of the 9,386 identified proteins 5,834 unique proteins were further quantified (**S1 Table,** *tab (a)***).** The TMT method showed significant bias toward host proteins and yielded significantly lower numbers of quantified proteins (452 vs. 3,007 quantified by PEAKS, **Table 1**). Notably, more host proteins are reduced in abundance upon resistant starch supplementation (*black lanes* indicate host proteins, while grey indicate bacterial proteins on the annotation bar next to the heatmap in **Fig 3,** *left*). This result is consistent with previous reports that indicate an increase in bacterial biomass upon resistant starch supplementation [3].

**Table 1:** Number of quantified proteins by various methods. For a protein to be quantified, a p-value had to be calculated (requiring at least 3 non-zero quantitative values in either CKD-DS or CKD-RS group). Total number of proteins quantified, and number of significant proteins using either p<0.05 or p<0.01 thresholds after Benjamini-Hochberg correction for multiple testing is reported for each of the quantification methods used.

Protein filter	TMT	PEAKS (NSAF)	PEAKS (Raw uSPC)	MaxQuant (NSAF)	MaxQuant (iBAQ)
All quantified	452	3007	3007	2843	2843
p.adj < 0.05	145	1198	1168	1208	1163
p.adj < 0.01	75	645	524	725	673

Hierarchical clustering procedures applied to abundance values derived by metaproteomics separated the two phenotypes into two distinct clusters (**Fig 3**, *left heatmap*). This separation also held when all proteins were considered, not just differentially abundant ones (**S3 Fig**). We note that that if rats **9** (CKD group) and **21** (CKD-RS group), were included in the heatmap, it would break the clear separation of the two phenotypes and form a separate cluster (**S4 Fig).** Upon further scrutiny, these two samples showed significant degradation of bacterial proteins, and as a consequence, low-quality fragmentation mass spectra and high host-to-bacterial protein ratio (**S2 Fig**). These samples were therefore excluded and not used for the final analysis.

There were 179 host proteins that were differentially abundant between the cecal contents of CKD-DS and CKD-RS rats: 125 were proteins reduced in CKD-RS and 54 increased in CKD-RS (**Table 2**, **S1 Table–**complete protein list). Among the 125 host proteins that had lower abundances in CKD RS, approximately half were enzymes. The remaining proteins were related to humoral immune response, several proteins previously associated with epithelial–mesenchymal transition (e.g. thioredoxin, S100-A6) and several proteins, previously reported, directly or indirectly, to be associated with CKD (**Table 2**).

**Table 2:** Representative differentially-abundant host proteins in cecal contents from CKD rats fed digestible starch (DS) or resistant starch (RS).

Rat Proteins	Log2(CKD RS-fed: CKD)^a^	p value^b^	cited to be CKD-related
***reduced***			
thioredoxin	-3.2, -1.7, -1.1, -1.5	2x10^-3^	
A1-microglobulin	-3.9, nd, nd, nd,	6x10^-10^	[30]
haptoglobin	nd, nd, -2.3, -2.7	5 x 10^−3^	[30, 31]
calcineurin	-3.0, -3.4, nd, nd	1.8 x 10^−5^	[32]
S100-A6	Nd, -2.6, -3.7, -7.1		
calreticulin	-1.6, -3.9, -3.6, -6.1	3x10^-5^	[33]
Angiotensin-converting enzyme	nd, nd, -2.5, -4.3	1x10^-3^	Well-established therapeutic target in CKD[34]
***increased***			
Annexins A7, VII,A2	2 to 3 ^c^	10^−3^ to 10^−8^ ^c^	[35]
voltage-dependent anion-selective channel protein 2	1.6, 4.5, 2.8, 5.2	2x10^-6^	[36, 37]
Claudin-3	nd, nd, 2.0, 3.2	9x10^-3^	

^a^ logarithm of ratio between abundance values between RS-fed and DS-fed groups. Four values are reported, corresponding to four different methods of analysis in the following order: TMT-labeling, PEAKS spectral counting (NSAF), MaxQuant spectral counting (NSAF), and MaxQuant iBAQ, *nd*–not determined

^b^minimal p-value across all four methods derived using moderated t-test, corrected for multiple testing.

^c^range across proteins in the same family.

Among the 54 host proteins that were increased in abundances in the CKD-RS group, about one third were enzymes. The largest group of proteins with similar functions was related to the immunoglobulin families. Another group included annexins (**Table 2**, **S1 Table)** that function as intracellular Ca^2+^ sensors and participate in cellular membrane repair [35], suggesting that in CKD-RS rats a process of intestinal cell repair, presumably damaged by bacteria and/or bacterial metabolites, is ongoing. Other small groups of proteins with similar function included a group of voltage-dependent ion channel proteins that regulate fluxes across the outer mitochondrial membrane and sodium pump subunit proteins. Mitochondrial impairment has been shown in CKD patients and animal models, particularly in the form of a decrease in mitochondrial DNA and down-regulation of many mitochondrial genes and proteins [36, 37]. Down-regulation of voltage channel proteins in CKD rats could be, in part, causing the increase of oxidative stress in CKD. Similar to previous studies [3, 16], we observed an increase in the tight junction protein claudin-3 (2.6 fold change in CKD-RS).

Interestingly, many of the individual proteins we found to be differentially abundant between CKD-DS and CKD-RS rats were previously identified in other reports on CKD and kidney associated diseases [16, 30, 32, 33, 37, 38]. Importantly, changes in these proteins were previously identified either in plasma or in urine. Identification of these proteins in cecal contents points to a potential utility of stool samples as a source of CKD protein biomarkers; however, the pathophysiological link between the association of the proteins in the GI tract with resistant starch and their association in urine and plasma during CKD is unclear.

### Molecular processes influenced by dietary resistant starch supplementation in the cecal contents of CKD rats

To define enrichment of functional patterns associated with the differentially abundant proteins found by metaproteomics, we performed Gene Ontology analysis using Blast2Go [39]. There were 179 differentially-abundant host proteins described above, as well as 1198 differentially abundant bacterial proteins, the latter of which included 359 reduced and 839 higher proteins with RS feeding. The list of proteins differentially abundant between CKD-DS and CKD-RS rats was tested for enrichment of GO terms (biological process, molecular function and cellular component) for bacterial and host proteins separately using Fisher exact test. All GO terms significant at the FDR level of 0.05 were considered significantly enriched.

#### GO categories, under-represented in CKD-RS samples for rat proteins

GO categories under-represented in the CKD-RS list of rat proteins (**Table 3**) were related to known biological processes occurring in CKD, such as aldehyde metabolism (indicative of lipid peroxidation processes), and humoral immune response. Reactive aldehydes are predominantly generated as a result of oxidative stress-induced lipid peroxidation, a process elevated in CKD patients [40]. Therefore, reduction in aldehyde biosynthesis and catabolism categories in CKD-RS rats is likely a consequence of the ongoing lipid peroxidation process in CKD rats. Reduction in positive regulation of humoral immune response category in CKD-RS rats first indicates the presence of residual bodily fluids in feces and second, that antimicrobial immune response is activated in CKD rats. Response to nutrients category includes many enzymes with different activities that are involved simultaneously in many GO biological processes, so it is difficult to dissect specific CKD-RS related process resulting in reduction ‘response to nutrients’ category. Two most specific molecular functions, reduced in CKD-RS rats were serine-type endopeptidase inhibitor and metalloexopeptidase activities. Only one cellular component category was reduced in CKD-RS rats, namely membrane-bounded organelle, showing that processes activated in CKD rats involve cellular membranes.

**Table 3:** Gene ontology categories under-represented in the CKD-RS host proteins.

GO Name	GO Cat	FDR	p value	Nr Test	Nr Ref	Non Annot Test	Non Annot Ref
organic substance metabolic process	B.P.	0.001004	1.71E-07	111	637	17	339
metabolic process	B.P.	0.001004	3.24E-07	115	684	13	292
primary metabolic process	B.P.	0.001376	6.66E-07	106	601	22	375
cellular metabolic process	B.P.	0.007216	4.66E-06	93	506	35	470
cellular aldehyde metabolic process	B.P.	0.00905	7.31E-06	14	20	114	956
aldehyde biosynthetic process	B.P	0.014295	1.38E-05	6	1	122	975
aldehyde catabolic process	B.P.	0.018199	2.15E-05	7	3	121	973
response to nutrient	B.P.	0.018199	2.35E-05	14	23	114	953
positive regulation of humoral immune response	B.P.	0.034493	5.01E-05	6	2	122	974
serine-type endopeptidase inhibitor activity	M.F.	0.008789	1.33E-05	15	25	113	951
catalytic activity	M.F.	0.008789	6.67E-06	89	477	39	499
metal ion binding	M.F.	0.039583	1.77E-04	56	269	72	707
hemoglobin binding	M.F.	0.039583	1.73E-04	4	0	124	976
endopeptidase inhibitor activity	M.F.	0.039583	2.39E-04	18	48	110	928
identical protein binding	M.F.	0.039583	1.06E-04	37	144	91	832
peptidase inhibitor activity	M.F.	0.039583	2.39E-04	18	48	110	928
endopeptidase regulator activity	M.F.	0.039583	2.39E-04	18	48	110	928
peptidase regulator activity	M.F.	0.047892	3.61E-04	18	50	110	926
metalloexopeptidase activity	M.F.	0.047892	3.26E-04	10	16	118	960
cation binding	M.F.	0.049124	4.08E-04	56	278	72	698
membrane-bounded organelle	C.C.	0.030257	4.18E-05	119	773	9	203

GO categories as follows: B.P.–biological process; M.F.–molecular function; C.C.–cellular component

#### GO categories significantly over-represented in CKD-RS rat proteins

There were no biological processes significantly over-represented in CKD-RS rat proteins at the FDR significance level 0.05. Molecular functions, significantly over-represented in CKD-RS rat proteins (**Table 4)** were related to the sodium-potassium pump subunits and cross-membrane transport; this may indicate that control of pH, osmotic pressure and cell volume are compromised in CKD rats and improve with the RS-rich diet. The most specific cellular components over-represented in CKD-RS rats were zymogen granule membrane, sodium:potassium-exchanging ATPase complex and extracellular exosome. All these molecular functions and cellular compartments were over-represented because of the presence of several specific proteins in CKD-RS differentially abundant protein list, but the amount of these proteins was not enough to identify enriched biological processes at the given significance level. However, if instead of FDR 0.05 we would consider *p*-value based significance level 0.001 then five biological processes would become significant: cellular sodium ion homeostasis, cellular potassium ion homeostasis, sodium ion export from cell, potassium ion import across plasma membrane and relaxation of cardiac muscle. All these categories were present because of the several sodium pump subunit proteins in CKD-RS DA protein list. However, the mechanisms of sodium-potassium pump regulation are complicated: for example, it was suggested that cardiotonic steroids mediate signal transduction through the Na/K-ATPase, and its downregulation could be indirectly implicated in profibrotic pathways [41]. Given that it is difficult to predict the consequences of insufficient sodium pump levels without further experiments, this observation warrants future study.

**Table 4:** Gene ontology categories over-represented in the CKD-RS host proteins.

GO Name	GO Cat	FDR	p value	Nr Test	Nr Ref	Non Annot Test	Non Annot Ref
calcium-dependent phospholipid binding	M.F.	0.001064	8.02E-07	7	8	40	1049
calcium ion binding	M.F.	0.005162	1.17E-05	14	79	33	978
calcium-dependent protein binding	M.F.	0.005162	1.00E-05	6	8	41	1049
phospholipid binding	M.F.	0.015317	4.62E-05	8	26	39	1031
lipid binding	M.F.	0.026549	1.00E-04	12	72	35	985
sodium:potassium-exchanging ATPase activity	M.F.	0.045896	3.12E-04	4	5	43	1052
sodium ion binding	M.F.	0.045896	3.12E-04	4	5	43	1052
sodium ion transmembrane transporter activity	M.F.	0.045896	3.12E-04	4	5	43	1052
potassium-transporting ATPase activity	M.F.	0.045896	3.12E-04	4	5	43	1052
membrane	C.C.	8.39E-10	1.16E-12	46	532	1	525
membrane part	C.C.	2.27E-08	6.28E-11	39	372	8	685
whole membrane	C.C.	1.72E-07	7.15E-10	24	132	23	925
plasma membrane	C.C.	3.70E-07	2.04E-09	37	367	10	690
cell periphery	C.C.	1.15E-06	7.96E-09	37	384	10	673
plasma membrane part	C.C.	2.02E-06	1.67E-08	30	252	17	805
membrane microdomain	C.C.	3.34E-06	3.69E-08	15	55	32	1002
membrane raft	C.C.	3.34E-06	3.69E-08	15	55	32	1002
integral component of membrane	C.C.	4.60E-06	5.72E-08	25	182	22	875
intrinsic component of membrane	C.C.	1.53E-05	2.12E-07	25	195	22	862
membrane region	C.C.	3.90E-05	5.92E-07	15	70	32	987
endomembrane system	C.C.	3.96E-05	6.57E-07	27	241	20	816
bounding membrane of organelle	C.C.	5.19E-05	9.31E-07	17	96	30	961
integral component of plasma membrane	C.C.	9.20E-05	1.78E-06	12	46	35	1011
intrinsic component of plasma membrane	C.C.	2.62E-04	5.44E-06	12	52	35	1005
extracellular region part	C.C.	6.60E-04	1.46E-05	47	829	0	228
secretory vesicle	C.C.	8.30E-04	1.95E-05	14	83	33	974
intracellular vesicle	C.C.	0.001095	2.87E-05	20	171	27	886
cytoplasmic vesicle	C.C.	0.001095	2.87E-05	20	171	27	886
secretory granule membrane	C.C.	0.001141	3.22E-05	7	17	40	1040
extracellular region	C.C.	0.001141	3.31E-05	47	844	0	213
plasma membrane region	C.C.	0.00152	4.62E-05	16	117	31	940
zymogen granule	C.C.	0.001712	5.44E-05	6	12	41	1045
sodium:potassium-exchanging ATPase complex	C.C.	0.002472	9.22E-05	4	3	43	1054
ATPase complex	C.C.	0.002472	9.22E-05	4	3	43	1054
ATPase dependent transmembrane transport complex	C.C.	0.002472	9.22E-05	4	3	43	1054
cation-transporting ATPase complex	C.C.	0.002472	9.22E-05	4	3	43	1054
vesicle	C.C.	0.003119	1.21E-04	44	743	3	314
secretory granule	C.C.	0.004072	1.63E-04	11	64	36	993
apical plasma membrane	C.C.	0.004847	2.01E-04	12	78	35	979
organelle membrane	C.C.	0.008826	3.78E-04	18	173	29	884
pore complex	C.C.	0.015435	6.82E-04	3	2	44	1055
zymogen granule membrane	C.C.	0.016801	7.66E-04	4	7	43	1050
sarcolemma	C.C.	0.018682	8.77E-04	7	32	40	1025
caveola	C.C.	0.023022	0.001113	4	8	43	1049
apical part of cell	C.C.	0.025281	0.001257	12	97	35	960
plasma membrane raft	C.C.	0.029032	0.001558	4	9	43	1048
cytoplasmic vesicle part	C.C.	0.029032	0.001549	9	59	38	998
vesicle membrane	C.C.	0.029032	0.001564	8	47	39	1010
cell part	C.C.	0.031352	0.00177	46	875	1	182
specific granule	C.C.	0.031352	0.001775	2	0	45	1057
cell	C.C.	0.031965	0.001854	46	876	1	181
phagocytic vesicle	C.C.	0.037838	0.002247	3	4	44	1053
extracellular exosome	C.C.	0.043258	0.002868	41	721	6	336
extracellular vesicle	C.C.	0.043258	0.002868	41	721	6	336
transmembrane transporter complex	C.C.	0.043258	0.002792	4	11	43	1046
extracellular organelle	C.C.	0.043258	0.002868	41	721	6	336
transporter complex	C.C.	0.043258	0.002792	4	11	43	1046
nuclear envelope	C.C.	0.046915	0.003175	6	30	41	1027

GO categories as follows: B.P.–biological process; M.F.–molecular function; C.C.–cellular component

Many biological processes and molecular functions, associated with CKD, especially at the more advanced stages, were previously characterized by transcriptomics. These included down-regulation in the kidney of regulatory proteins involved in cytoskeleton organization, microtubule assembly and stability, epithelial–mesenchymal transition, extracellular matrix remodeling, cell motility and migration, cell adhesion, apoptosis, cell differentiation, proteolysis, aminoglycan metabolic process and protein N-linked glycosylation [42, 43]. One advantage metaproteomics offers is the simultaneous analysis of bacterial and host proteins, with metaproteomics offering higher resolution of the bacterial portion of the proteome compared to metatranscriptomics. Indeed, in its current stage dual transcriptomics (i.e. simultaneous analysis of host and microbiome transcripts) is more challenging than metaproteomics. The pipeline for dual RNA-seq analysis has the same building blocks as conventional RNA-seq pipeline: reads must be cleaned, mapped, normalized and differentially expressed transcripts identified. However, as recommended by Avraham et al [44] host and pathogen reads should be mapped to the reference genomes and analyzed separately [44]. The latter creates a difficulty of using additional READemption pipeline for mapping bacterial reads, in addition to conventional TopHat pipeline for mapping host reads. READemtion pipeline uses the short read mapper segemehl and its remapper lack [45, 46] unlike Tophat that uses bowtie [47]. Also, when ones choses between transcriptomics and proteomics to characterize a disease–in our opinion proteomics is a better choice because 1) cellular phenotypes are defined by proteins more so than by transcripts, hence proteins are better biomarkers 2) druggable targets are usually proteins and not transcripts, and 3) correlation between protein abundance and transcript abundance is typically 40% to 60% depending on the cell type.

The analysis of significantly under-represented GO categories in CKD-RS for bacterial proteins supports the idea that in CKD-DS samples there is an ongoing process of mucin degradation that is more active than in CKD-RS samples (**Table 5**), in particular monosaccharide metabolic process and L-fucose catabolic process are significant. These processes may refer to the way bacteria in CKD rats preferably degrade mucins, containing fucosyl residues at the extremity of O-glycosidic chain [48]. The importance of this metabolic process is confirmed by the presence of fucosidase-encoding genes in the list of DA proteins (S1 Table). The idea of preferential foraging on mucin proteins by gut microbiota in CKD-DS rats is further supported by the list of bacterial species, under-represented in CKD-RS, where several mucin degraders are evident (e.g. *Mucispirillum schaedleri*, *R*.*gnavus*, *R*.*torques–***S2 Table**).

**Table 5:** GO categories under-represented in CKD-RS microbial proteins.

GO Name	GO Cat	FDR	P-Value	Nr Test		Nr Ref		Non Annot Test		Non Annot Ref	
metabolic process	B.P.	3.62E-04	3.20E-07		243		3127		48		1320
small molecule catabolic process	B.P.	0.006091	1.08E-05		24		126		267		4321
single-organism process	B.P.	0.011151	3.77E-05		173		2104		118		2343
small molecule metabolic process	B.P.	0.011151	3.95E-05		126		1410		165		3037
monosaccharide metabolic process	B.P.	0.013406	8.77E-05		40		314		251		4133
single-organism metabolic process	B.P.	0.013406	8.40E-05		157		1887		134		2560
fucose catabolic process	B.P.	0.013406	1.19E-04		7		13		284		4434
organic substance metabolic process	B.P.	0.013406	1.11E-04		195		2485		96		1962
L-fucose metabolic process	B.P.	0.013406	1.19E-04		7		13		284		4434
L-fucose catabolic process	B.P.	0.013406	1.19E-04		7		13		284		4434
cellular metabolic process	B.P.	0.027933	2.72E-04		172		2156		119		2291
primary metabolic process	B.P.	0.038022	4.04E-04		179		2279		112		2168
hexose catabolic process	B.P	0.038472	4.43E-04		10		37		281		4410

GO categories as follows: B.P.–biological process; M.F.–molecular function; C.C.–cellular component

#### GO categories over-represented in CKD-RS microbial proteins

For bacterial proteins, GO categories over-represented in CKD-RS samples (biological processes, molecular functions and cellular components, **Table 6**.) were all related to active bacterial proliferation, emphasizing a shift in CKD-RS microbiota toward new actively dividing bacterial populations, which are able to thrive on RS.). Active bacterial proliferation can also partially explain the previously observed reduction in harmful tryptophan and tyrosine metabolites in CKD-RS rats (indoxyl and p-cresol, respectively). Instead of conversion to the toxins, tryptophan and tyrosine could be incorporated into newly synthesized proteins.

**Table 6:** GO categories over-represented in CKD-RS microbial proteins.

GO Name	GO Cat	FDR	P-Value	Nr Test	Nr Ref	Non Annot Test	Non Annot Ref
structural constituent of ribosome	M.F.	3.62E-05	8.06E-08	94	270	641	3733
catalytic activity	M.F.	3.62E-05	4.33E-08	536	2511	199	1492
binding	M.F.	4.52E-05	1.51E-07	434	1950	301	2053
rRNA binding	M.F.	1.52E-04	6.75E-07	68	181	667	3822
organic cyclic compound binding	M.F.	0.002196	1.95E-05	318	1408	417	2595
RNA binding	M.F.	0.002196	1.57E-05	110	383	625	3620
heterocyclic compound binding	M.F.	0.002196	1.95E-05	318	1408	417	2595
nucleic acid binding	M.F.	0.002196	1.59E-05	127	460	608	3543
molecular transducer activity	M.F.	0.00337	3.37E-05	21	34	714	3969
receptor activity	M.F.	0.00772	8.59E-05	20	34	715	3969
oxidoreductase activity, acting on the CH-OH group of donors, NAD or NADP as acceptor	M.F.	0.032212	3.94E-04	53	166	682	3837
oxidoreductase activity, acting on CH-OH group of donors	M.F.	0.034521	4.99E-04	53	168	682	3835
hydrolase activity	M.F.	0.034521	4.95E-04	126	499	609	3504
metabolic process	B.P.	2.49E-14	2.20E-17	614	2756	121	1247
primary metabolic process	B.P.	2.17E-07	3.85E-10	458	2000	277	2003
organic substance metabolic process	B.P.	3.03E-07	8.06E-10	490	2190	245	1813
macromolecule metabolic process	B.P.	2.08E-06	7.37E-09	204	733	531	3270
protein metabolic process	B.P.	5.76E-06	2.95E-08	175	613	560	3390
cellular metabolic process	B.P.	5.76E-06	3.06E-08	429	1899	306	2104
cellular nitrogen compound metabolic process	B.P.	9.55E-06	5.92E-08	280	1126	455	2877
cellular macromolecule metabolic process	B.P.	3.13E-05	2.22E-07	172	620	563	3383
cellular protein metabolic process	B.P.	4.40E-05	3.50E-07	148	515	587	3488
cellular biosynthetic process	B.P.	8.03E-05	7.31E-07	242	970	493	3033
peptide metabolic process	B.P.	8.03E-05	7.83E-07	142	496	593	3507
cellular nitrogen compound biosynthetic process	B.P.	9.09E-05	9.66E-07	191	726	544	3277
translation	B.P.	1.22E-04	1.63E-06	139	490	596	3513
biosynthetic process	B.P.	1.22E-04	1.59E-06	273	1136	462	2867
organic substance biosynthetic process	B.P.	1.22E-04	1.73E-06	268	1112	467	2891
peptide biosynthetic process	B.P.	1.22E-04	1.63E-06	139	490	596	3513
gene expression	B.P.	2.48E-04	3.73E-06	151	554	584	3449
cellular macromolecule biosynthetic process	B.P.	6.18E-04	1.09E-05	150	562	585	3441
macromolecule biosynthetic process	B.P.	6.18E-04	1.09E-05	150	562	585	3441
organonitrogen compound biosynthetic process	B.P.	6.18E-04	1.08E-05	208	840	527	3163
nitrogen compound metabolic process	B.P.	9.42E-04	1.75E-05	326	1448	409	2555
amide biosynthetic process	B.P.	0.001246	2.43E-05	140	525	595	3478
organonitrogen compound metabolic process	B.P.	0.001384	2.82E-05	302	1331	433	2672
cellular amide metabolic process	B.P.	0.001969	4.19E-05	146	560	589	3443
cellular process	B.P.	0.004338	9.61E-05	476	2296	259	1707
organophosphate metabolic process	B.P.	0.00609	1.40E-04	119	449	616	3554
phospholipid metabolic process	B.P.	0.006387	1.58E-04	11	11	724	3992
phospholipid biosynthetic process	B.P.	0.006387	1.58E-04	11	11	724	3992
carbohydrate derivative metabolic process	B.P.	0.016781	4.31E-04	115	445	620	3558
ribose phosphate metabolic process	B.P.	0.036465	9.69E-04	99	381	636	3622
cytoplasmic part	C.C.	6.24E-07	1.42E-08	110	323	625	3680
intracellular ribonucleoprotein complex	C.C.	6.24E-07	2.13E-08	97	273	638	3730
ribosome	C.C.	6.24E-07	1.84E-08	97	272	638	3731
ribonucleoprotein complex	C.C.	6.24E-07	2.13E-08	97	273	638	3730
macromolecular complex	C.C.	7.06E-07	3.02E-08	118	362	617	3641
intracellular non-membrane-bounded organelle	C.C.	7.49E-07	3.84E-08	97	277	638	3726
intracellular organelle	C.C.	9.89E-07	5.91E-08	97	280	638	3723
outer membrane	C.C.	9.45E-04	6.46E-05	15	19	720	3984
intracellular	C.C.	0.001975	1.52E-04	287	1284	448	2719
intracellular part	C.C.	0.002387	2.24E-04	284	1276	451	2727
cell envelope	C.C.	0.002387	2.06E-04	15	22	720	3981
envelope	C.C.	0.002448	2.93E-04	15	23	720	3980
cytoplasm	C.C.	0.002448	2.69E-04	281	1264	454	2739
ribosomal subunit	C.C.	0.002448	2.72E-04	29	69	706	3934
membrane	C.C.	0.002964	3.80E-04	59	191	676	3812
intracellular organelle part	C.C.	0.004174	5.71E-04	29	73	706	3930
cell outer membrane	C.C.	0.009037	0.00139	11	16	724	3987
external encapsulating structure part	C.C.	0.009037	0.00139	11	16	724	3987
small ribosomal subunit	C.C.	0.014985	0.002433	14	27	721	3976
cell	C.C.	0.017721	0.003029	321	1529	414	2474
cell part	C.C.	0.03902	0.007004	314	1514	421	2489

GO categories as follows: B.P.–biological process; M.F.–molecular function; C.C.–cellular component

### Cecal microbiome composition revealed by metaproteomics at high resolution

#### Alpha-diversity

Using metaproteomics data for the inference of taxonomic units, we observed increases in alpha diversity for the samples derived from the CKD-RS rats, when the lowest level of taxonomy is considered. iBAQ and SAF methods of quantification give a 50% (p = 0.013) and 30% increase (p = 0.008), respectively (**S5 Fig**). This increase in alpha diversity is opposite to what has been reported in previous studies of resistant starch supplementation in healthy pigs where 16S RNA was used for the taxonomic inference [11]; it is also opposite to what has been inferred from the same rats in Kieffer et al. using 16S RNA [5]. The reason for this discrepancy has to do with taxonomic resolution: the diversity drops when only family and genus taxonomic levels are considered (limit of resolution for 16S RNA method), but the diversity within specific families increases at the species level (see example for *Ruminococcus* genus below). Thus, our data using metaproteomics support an increase in microbial diversity in response to RS diet.

#### Changes in gut microbial composition

As with the 16S RNA studies, we observe an increase in the *Bacteroidetes*-to-*Firmicutes* ratio in CKD-RS group. However, because of the species-level resolution the absolute numbers differ. In the previous analysis using 16S RNA, an overall ~2.5 times increase in *Ruminococcus genus* was observed. Metaproteomics analysis provides species-level resolutions and as a result, some species of *Ruminococcus* dramatically increase and some dramatically decrease with the RS supplementation. For example, *Ruminococcus bromii* is the key degrader of resistant starch in the mammalian gut [12]. According to the metaproteomics analysis (**S1 Table**, *tab (c)*, **S2 Table**), *Ruminococcus bromii L2-63* is increased ~12 times upon addition of resistant starch. At the same time, some species of this genus were decreased. For example, *Ruminococcus albus* decreased 20 fold and *Ruminococcus flavefaciens* decreased 10 fold. *R*. *albus*, *R*.*gnavus* and *R*.*flavefaciens* are fibrolytic bacteria that are able to process complex plant polysaccharides by their cellulolytic and hemicellulolytic enzymes. They use ammonia almost exclusively as their source of nitrogen. These bacteria are also known to thrive under high pH (above 6.0). Concordantly, in the preceding study pH was shown to drop from 8 to 6.75 in the CKD-RS rats [5, 16]. In contrast, *R*. *bromii* and *R*.*torques* are amylolytic and use RS as the main source of nutrients. Amylolytic (resistant starch degraders) and fibrolytic (cellulose degraders) species split the *Ruminoccocus* genus into two groups. Strikingly, the taxonomic resolution of metaproteomics analysis allowed us to observe clearly that fibrolytic *Ruminoccocus* are reduced with dietary RS, while amylolytic species are increased with the addition of RS. Beyond the biological importance of this observation per se, it demonstrates the power of metaproteomics analysis and the lower resolution of the 16S RNA method.

### Potential mechanisms of RS action

The question that remains to be fully understood is the mechanism(s) through which the RS diet exerts such a dramatic shift in control of biological processes and functions that are different between CKD-DS and CKD-RS rats. In part, it might be explained by bacterial population that shifts from mucin foraging to RS as a source of nutrients, relieving the host system from the constant flow of toxins that traverse the gut barrier. Other effects may involve reduction of oxidative stress. In the past, it was shown that kidneys from CKD patients have an impaired mitochondrial respiratory system [37], decreased DNA mitochondrial copy number [36], loss of mitochondrial membrane potential and lower ATP production [49]. The hypothesis that new bacterial populations influence mitochondrial biogenesis and activity (for example, through the influx of short chain fatty acids) is an attractive one. Recently it was shown that butyrate supplementation improved mitochondrial biogenesis in mice [50], presumably via inhibition of histone deacetylase (HDAC) that may down-regulate expression of PGC-1α, associated with mitochondrial dysfunction [51–53]. There is growing evidence that RS prevents colonic DNA damage via the production of SCFA, especially butyrate [54, 55]. In fact, it has been known, for almost a decade, that butyrate has a central role in maintaining gut epithelial integrity via involvement in key biological processes, such as being a source of energy for colonocytes, promoting fatty acid oxidation, having anti-inflammatory activity, limiting oxidative stress and inducing cell cycle arrest [56, 57]. In all these processes butyrate putatively functions by blocking substrate access to active sites in HDACs. Butyrate is a microbial fermentation product and butyrate producers are polyphyletic, belonging to different bacterial species, most known are members of *Lachnospiraceae* and *Ruminococcaeae* [58]. Butyrate is synthesized by those microorganisms via pyruvate and acetyl-coenzyme A (CoA) by breakdown of complex polysaccharides (such as RS). We observed that RS supplementation, at least for the *Ruminococcaeae* family, results in a shift from fibrolytic family members to amylolytic family members that are well known butyrate-producers (*Ruminococcus bromii*, *Ruminococcus torques)*. Typically, in the absence of sufficient dietary fibers, commensal and pathogenic bacteria start to forage on mucin glycans to harness carbon and energy [59, 60]. While we did not examine if microbes lowered with RS feeding are mucin degraders we did find indirect evidence in support of that from GO categories reduced with RS feeding (monosaccharide metabolic process and L-fucose catabolic process). We therefore hypothesize that there is a relative shortage of fiber in CKD-DS and the RS diet shifts gut microbial communities, from bacteria foraging on mucins and ammonia and contributing to leaky gut phenotype to bacteria utilizing RS instead, and producing butyrate as a byproduct of fermentation.

Recently it was found that out of 3,184 sequenced bacterial genomes, mostly from the Human Microbiome Project, 225 were likely to be butyrate producers [58]. From the list of bacteria, up-regulated in CKD-RS, only *Eubacterium rectale*, *Clostridium botulinum and Lachnospiraceae bacterium* strains are present in this list of potential butyrate producers, presumably because rat and human gut microbiomes differ. It would be interesting to evaluate quantitatively the amount of mucin degraders and butyrate producers caused by the diet shift by genomics and transcriptomics, to reduce biases due to proteomics undersampling (e.g. bias against low abundance proteins).

**Fig 4:**
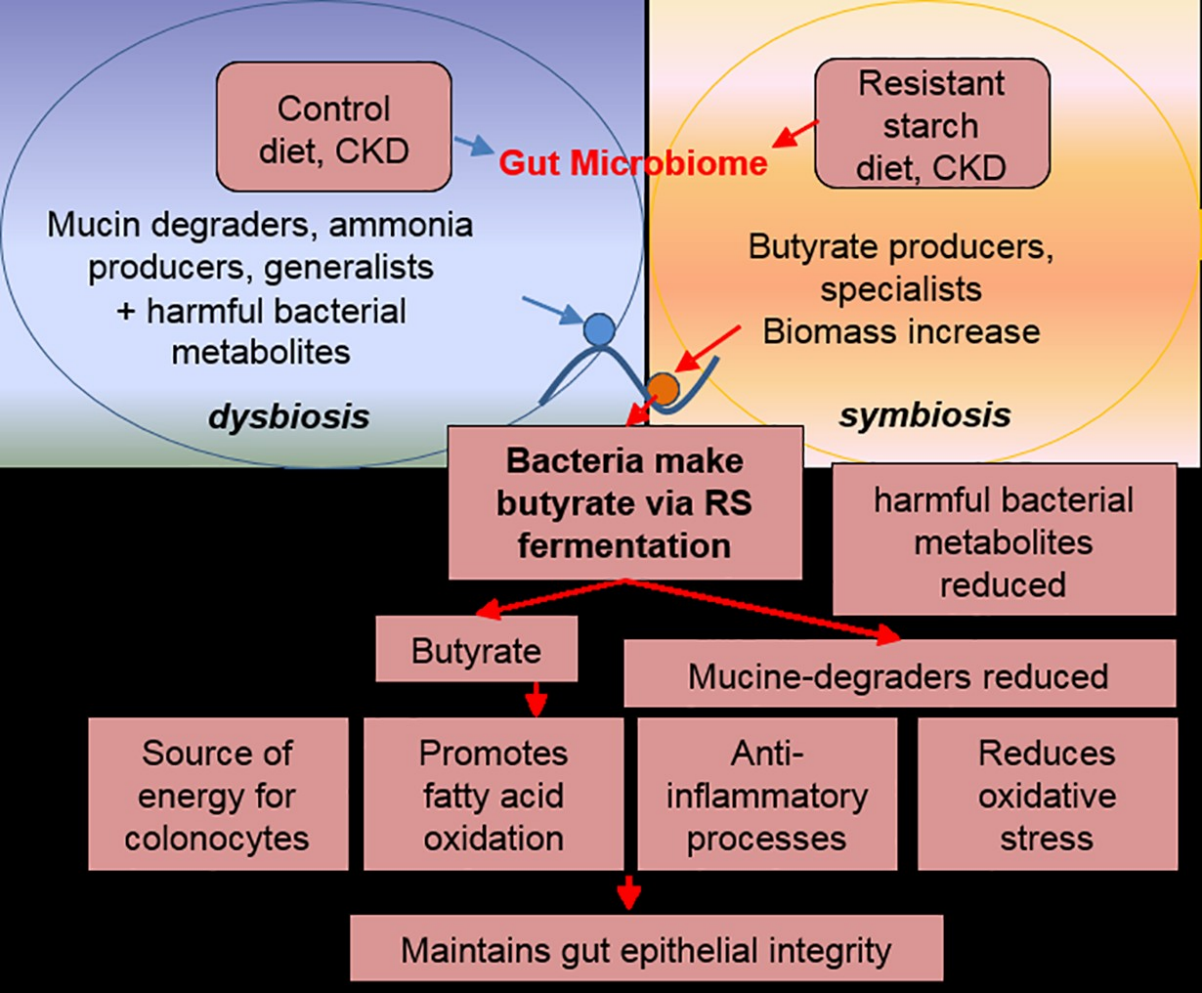
Working model of how RS diet changes gut microbiome composition, and in turn alleviates CKD. Dietary resistant starch supplementation results in a higher abundance of butyrate-producing bacteria and reduced abundance of mucin-degrading bacteria. Bacterially-derived butyrate mediates reduction of oxidative stress and inflammation. Integrity of gut epithelial barrier is also benefiting from reduced abundance of mucin-degrading bacteria. In addition, resistant starch supplementation leads to the active bacterial proliferation and the reduction of harmful bacterial metabolites, events that ameliorate CKD-induced oxidative stress and local and systemic inflammation. In fact, recent clinical trial has revealed that dietary supplementation of resistant starch, amylose, attenuates elevation of biomarkers of oxidative stress and inflammation in patients with end stage renal disease maintained on hemodialysis [61]-.

To summarize, dietary RS supplementation in rats ameliorates chronic kidney disease coincident with a massive shift in gut microbial communities (**Fig 4**). Identified organisms and proteins point toward a higher abundance of butyrate-producing bacteria, and reduced abundance of mucin-degrading bacteria. It is suggested that the bacterially-derived butyrate leads to reduction of oxidative stress and inflammation, as well as to improvement in other biological processes that are impaired in CKD. It is also possible, that the function of the gut epithelial barrier is improved during the RS supplementation due to the reduced degradation of mucins. Finally, resistant starch supplementation leads to the active bacterial proliferation and the reduction of harmful bacterial metabolites. The fact that simple change of available source of nutrients (from ammonia/mucins to RS) leads to system-level changes underscores the importance of diet during disease management and highlights the potential role of the gut microbiome in disease progression.

## Supporting information

S1 Checklist (PDF)

S1 Fig Metaproteomics analysis with de novo peptides. Initial taxonomic analysis based on de novo tags gerated via PEAKS Suite and is shown on the figure. The taxonomy trees were built using UniPept–an online metaproteomics tool. Top row–CKD rats on control diet, bottom row–CKD rats with resistant starch supplemented diet. The sample quality is assessed via ratio of green (host) to blue (bacteria) circle areas at the superkingdom taxonomic level. Outliers–CKD9 and CKDRS21 –are evident, host and bacteria taxonomic levels are marked with green and blue asterisks, respectively.
(PDF)

S2 Fig Host-to-bacterial ratio in the analyzed samples. The bar graph shows host-to-bacterial ratio of numbers of identified proteins. Samples 9 and 21 are evident outliers.
(PDF)

S3 Fig Hierarchical clustering of all proteins identified in NSAF analysis. NSAF analysis assigned quantitative value to proteins that later were not used in quantitative analysis and were filtered out due to low quality of quantification. We show this unfiltered data on ~9,300 proteins here to illustrate that the whole set of identified proteins–and not just those of high quality matches used later for quantification–separates the two phenotypes into distinct clusters, + resistant starch, and +digestible starch.
(PDF)

S4 Fig Proteins quantified with tandem mass tags. The heatmap figure shows the two outliers–samples from 9 and 21 clustered together breaking the clear separation of the two phenotypes. These samples were excluded from the final analysis and models.
(PDF)

S5 Fig Alpha diversity calculated with two different methods. The bar graph shows alpha-diversity at the species level calculated based on spectral abundance factors (blue bars) and the sum of precursor intensities (orange bars). Overall increase in alpha-diversity at the species level is noticeable upon resistant starch supplementation.
(PDF)

S1 Table Quantified proteins. Each of the quantified proteins had at least one assigned adjusted p-value across experimental platforms that were used. In the case of TMT, PEAKS NSAF, Max Quant NSAF and MaxQuant iBAQ values moderated t-test was used. In the case of PEAKS Raw Unique Spectral counts–Poisson-Tweedy distribution test (package tweeDEseq -[62]) was used to infer the p-values. All p-values were recalculated to account for multiple hypothesis testing. Number of hypothesis in each test corresponded to number of proteins deemed quantifiable. Criteria for quantification were: > = 2 unique peptides, 5 or less zero values in at least one of the two conditions (PEAKS and MaxQuant datasets) and > = 2 unique peptides, 2 or less zero values across 4 of the pooled samples (TMT dataset).
(XLSX)

S2 Table Quantified taxonomic groups. Quantitative values for each taxonomic group were derived using several different methods–spectral abundance factor, by summing individual protein spectral counts for each taxonomic group; tandem mass tags–by summing reporter ion intensities, iBAQ–by summing individual protein precursor intensities. Each of the quantified taxonomic groups had at least one assigned p-value. All p-values were recalculated to account for multiple hypothesis testing.
(XLSX)
