# Supplementary figures and images for: Metaproteomics reveals potential mechanisms by which dietary resistant starch supplementation attenuates chronic kidney disease progression in rats
Source: PLoS One. 2019 Jan 30;14(1):e0199274. doi: 10.1371/journal.pone.0199274 (PMC6353070; doi:10.1371/journal.pone.0199274)

S1 Fig. Preliminary Analysis using de novo peptides

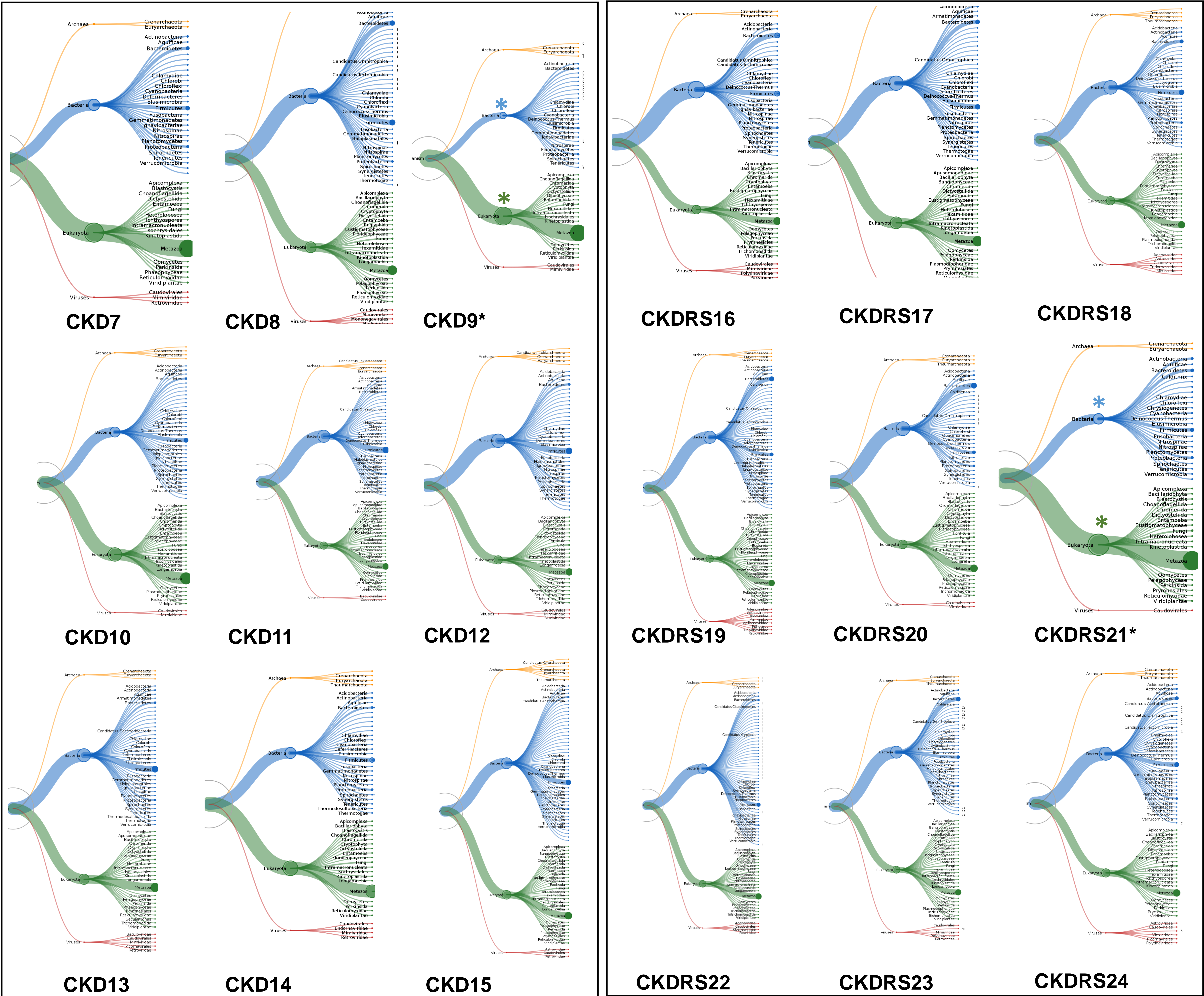

Supplement: S1 Fig — Initial taxonomic analysis based on de novo tags gerated via PEAKS Suite and is shown on the figure. The taxonomy trees were built using UniPept–an online metaproteomics tool. Top row–CKD rats on control diet, bottom row–CKD rats with resistant starch supplemented diet. The sample quality is assessed via ratio of green (host) to blue (bacteria) circle areas at the superkingdom taxonomic level. Outliers–CKD9 and CKDRS21 –are evident, host and bacteria taxonomic levels are marked with green and blue asterisks, respectively. (PDF) [file pone.0199274.s002.pdf]

S2 Fig. Host-to-bacterial ratio in the analyzed samples

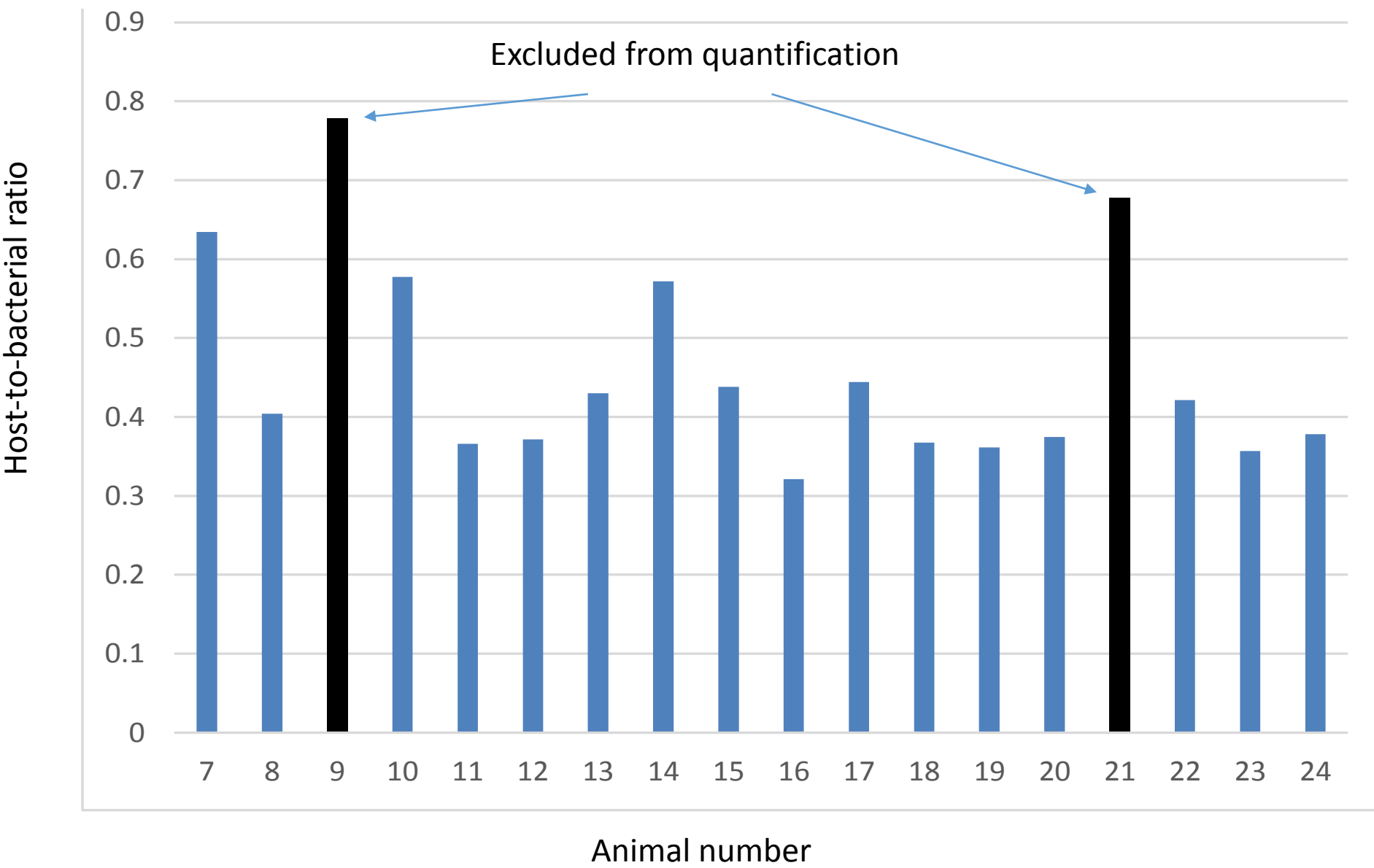

Supplement: S2 Fig — The bar graph shows host-to-bacterial ratio of numbers of identified proteins. Samples 9 and 21 are evident outliers. (PDF) [file pone.0199274.s003.pdf]

S5 Fig. Alpha diversity

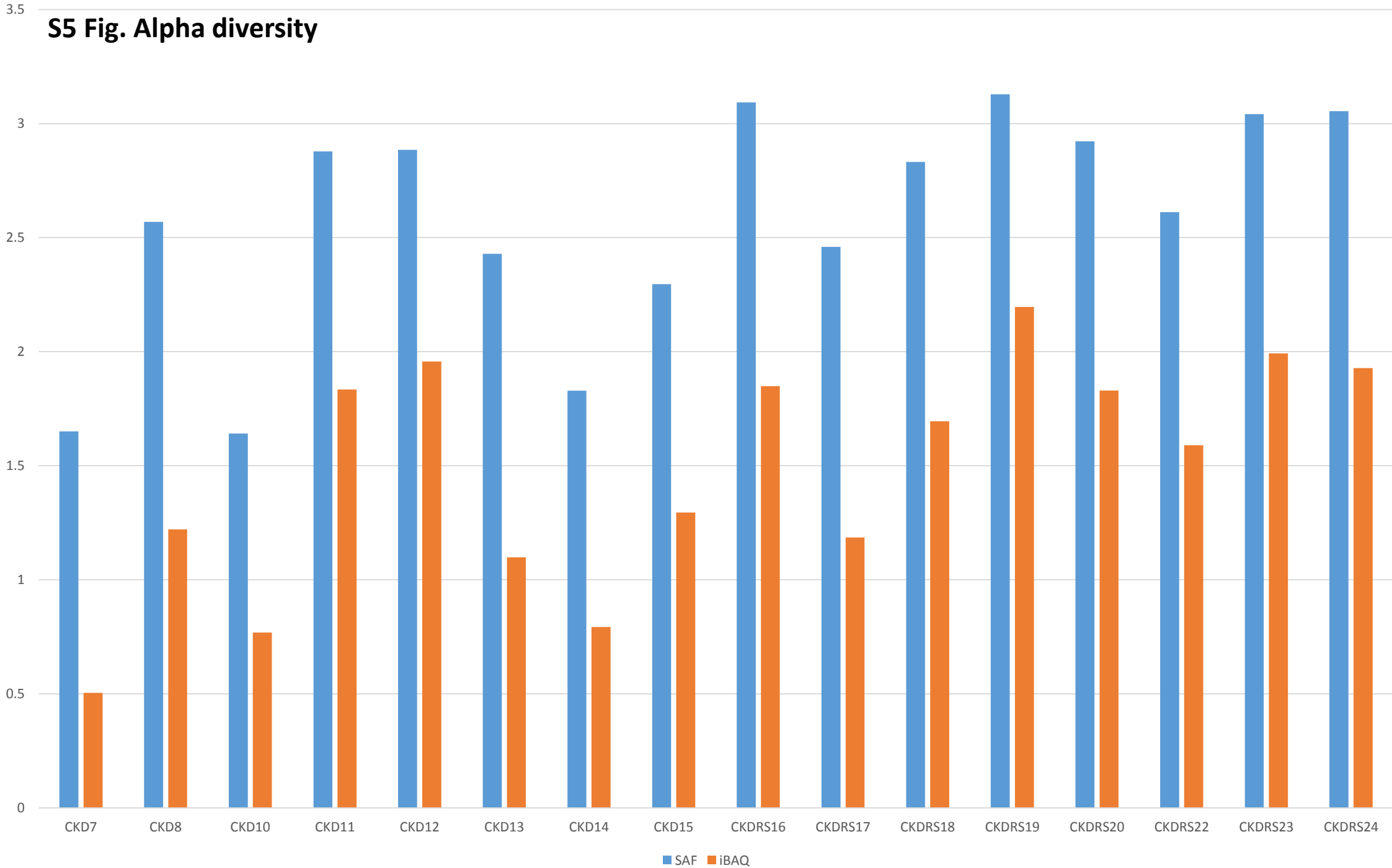

Supplement: S5 Fig — The bar graph shows alpha-diversity at the species level calculated based on spectral abundance factors (blue bars) and the sum of precursor intensities (orange bars). Overall increase in alpha-diversity at the species level is noticeable upon resistant starch supplementation. (PDF) [file pone.0199274.s006.pdf]
