# Supplementary material for: Metaproteomics reveals potential mechanisms by which dietary resistant starch supplementation attenuates chronic kidney disease progression in rats
Source: PLoS One. 2019 Jan 30;14(1):e0199274. doi: 10.1371/journal.pone.0199274 (PMC6353070; doi:10.1371/journal.pone.0199274)

S3 Fig. Hierarchical clustering of all proteins identified in NSAF analysis

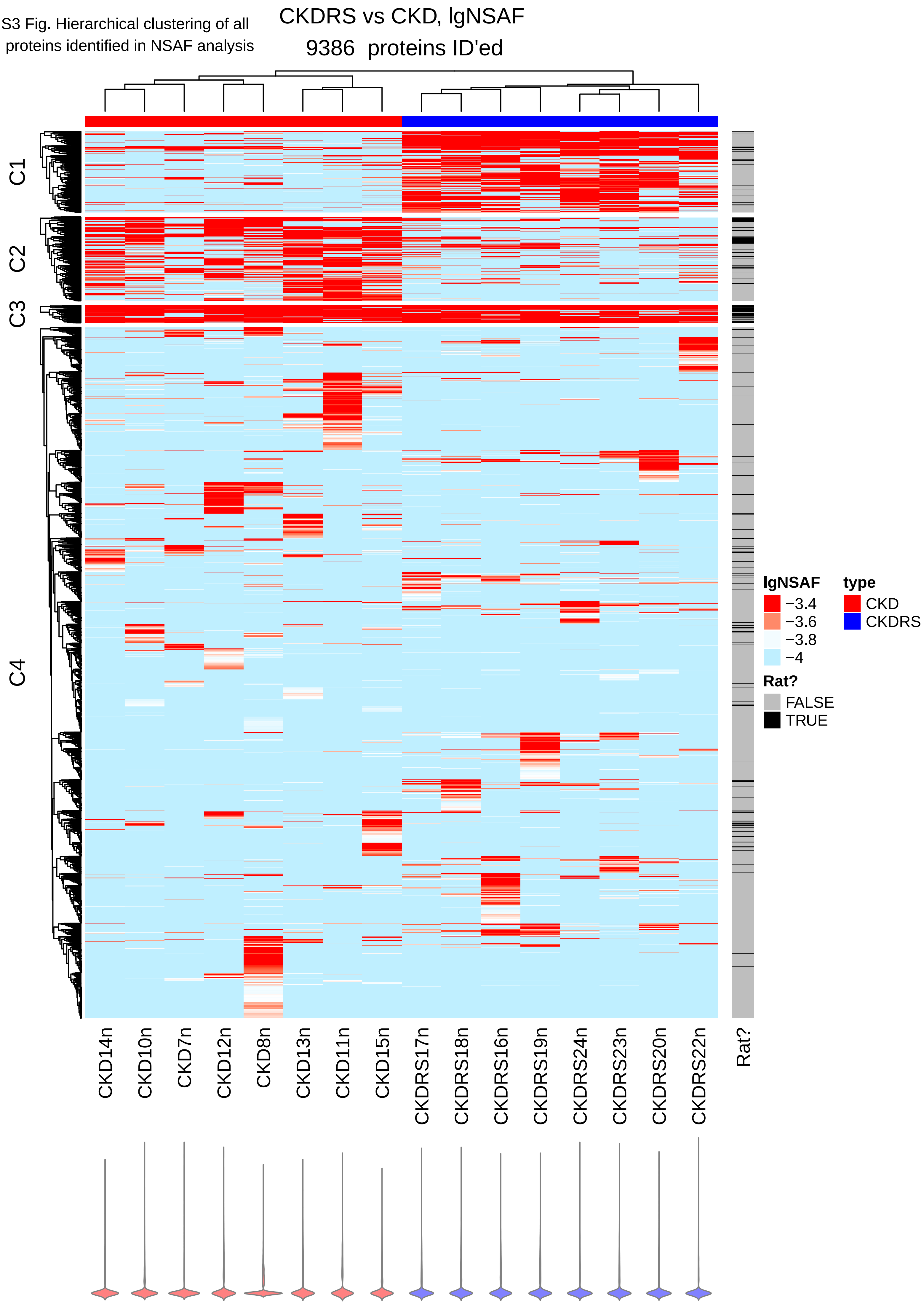

Supplement: S3 Fig — NSAF analysis assigned quantitative value to proteins that later were not used in quantitative analysis and were filtered out due to low quality of quantification. We show this unfiltered data on ~9,300 proteins here to illustrate that the whole set of identified proteins–and not just those of high quality matches used later for quantification–separates the two phenotypes into distinct clusters, + resistant starch, and +digestible starch. (PDF) [file pone.0199274.s004.pdf]
