# Supplementary material for: Metaproteomics reveals potential mechanisms by which dietary resistant starch supplementation attenuates chronic kidney disease progression in rats
Source: PLoS One. 2019 Jan 30;14(1):e0199274. doi: 10.1371/journal.pone.0199274 (PMC6353070; doi:10.1371/journal.pone.0199274)

S4 Fig. Proteins quantified with tandem mass tags.

Proteins quantified in TMT experiment with samples 9 and 21 included in the analysis

Samples from **Rats 9** and **21** break clear separation into two phenotypes

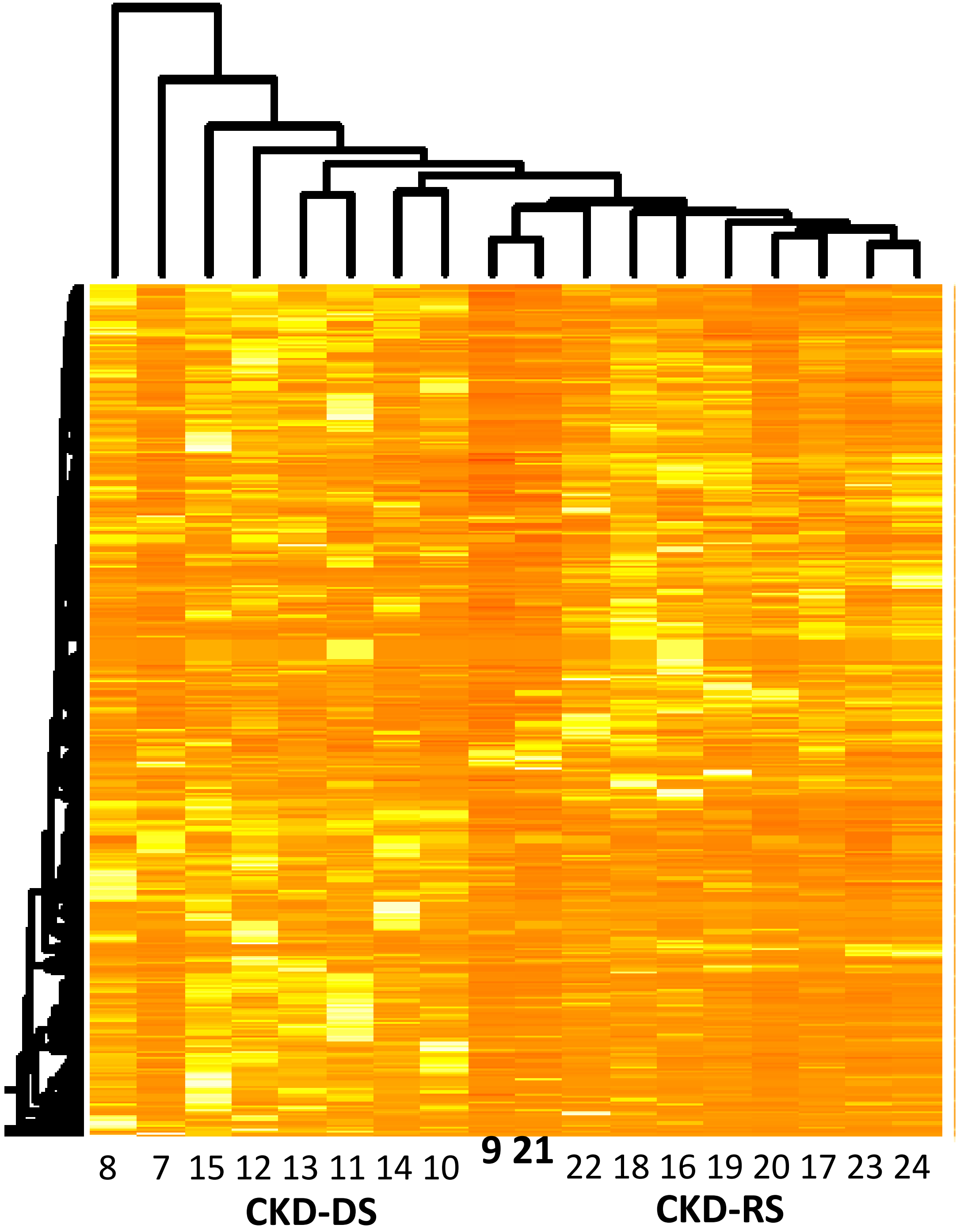

Supplement: S4 Fig — The heatmap figure shows the two outliers–samples from 9 and 21 clustered together breaking the clear separation of the two phenotypes. These samples were excluded from the final analysis and models. (PDF) [file pone.0199274.s005.pdf]
